# A Valid Approach in Refractory Glossodynia: A Single-Institution 5-Year Experience Treating with Japanese Traditional Herbal (Kampo) Medicine

**DOI:** 10.1155/2013/354872

**Published:** 2013-10-07

**Authors:** Hideki Okamoto, Atsushi Chino, Yoshiro Hirasaki, Keigo Ueda, Masaki Raimura, Takao Namiki

**Affiliations:** Japanese-Oriental (Kampo) Medicine, Chiba University Graduate School of Medicine, 1-8-1 Inohana, Chuo-Ku, Chiba 260-8670, Japan

## Abstract

Glossodynia is often refractory to conventional medicine, and there is only limited evidence to guide clinicians in its management. Patients with refractory glossodynia are often introduced to Japanese traditional herbal (Kampo) medicine experts under such circumstances because Kampo medicine has become known in Japan to be effective in treating a wide variety of symptoms refractory to conventional medicine. Herein, we report our single-institution 5-year experience treating patients with Kampo medicine for primary glossodynia that was refractory to conventional medicine. We found that 69.2% of patients reported a beneficial effect of Kampo medicine on glossodynia, and the average onset of improvement was 8.0 ± 7.7 weeks after starting Kampo treatment. The top two frequently used Kampo medicines for glossodynia were seinetsuhokito and mibakuekkito among high responders who showed a decrease of severity by 50% or more. The top four most overlapped herbs among effective Kampo medicines for glossodynia were Glycyrrhiza Root, Ginseng Root, Hoelen, and Atractylodes (lancea) Rhizome, which compose an essential Kampo prescription called shikunshito. Although more research is required to further clarify the effectiveness of Kampo medicine, it has valid efficacy even in cases of glossodynia that remain incurable by conventional treatments.

## 1. Introduction

Primary glossodynia, also known as glossopyrosis (burning tongue) or glossalgia (tongue pain), is characterized by chronic oral dysaesthesia in the setting of no identifiable clinical lesions, laboratory abnormalities, or causative systemic disease. Glossodynia is regarded as a type of burning mouth syndrome (BMS) that is often refractory to conventional medicine, and there is only limited evidence to guide clinicians in the management of patients with BMS. It is recommended that the treatment be tailored to each patient and that it be administered in a multidisciplinary facility [1]. The difficulty of treating BMS may result in BMS patients being unfavorably associated with a specific pattern of personality disorder comorbidity [2].

The Headache Classification Subcommittee of the International Headache Society defines BMS as “an intraoral burning sensation for which no medical or dental cause can be found” (Headache Classification Committee of the International Headache Society, 2004), and the International Association for the Study of Pain defines it as “a pain of at least 4–6 months duration located on the tongue or other mucosal membranes in the absence of clinical or laboratory findings.” Thus, BMS has been defined principally by the quality or location of the pain, and the diagnosis of BMS depends on the exclusion of a detectable organic basis for the complaint. A wide variety of pathogenic conditions therefore must be included such as local irritation; various mucocutaneous diseases; nutritional, metabolic, or endocrine disorders; xerostomia; and dysgeusia [3]. Kampo medicine, Japanese traditional herbal medicine, which originated in China, treats BMS based on Kampo-specific diagnostics, regardless of the pathogenesis determined on the basis of conventional medicine. 

The Japanese Ministry of Health, Labor, and Welfare has approved more than 210 Kampo prescriptions for clinical use in the same way that conventional medicines are prescribed. More than 80% of conventional medical doctors use Kampo medicine in their clinical practice, and most Kampo prescriptions are prescribed as freeze-dried extract granules. Freeze-dried Kampo-extract granules are manufactured by several pharmaceutical companies, and more than 140 Kampo prescriptions composed of those herbal extract granules are available for clinical use. Recently, Kampo medicine has received renewed attention because it provides a valid approach to treating symptoms refractory to conventional medicine and has become integrated with conventional medicine under the control of the Japanese Ministry of Health, Labor, and Welfare [4, 5].

We here focus on glossodynia, excluding pain in the other mucosal membranes, because the tongue is supposed to show the heart's condition in the five parenchymatous viscera theory in Kampo medicine, whereas the other parts of the mouth are not. There are some reports showing the effectiveness of alternative medicine for glossodynia, but no systematized medication with a high rate of effectiveness has been reported until now. Herein, we report our single-institution 5-year experience treating glossodynia with Kampo medicine.

## 2. Methods

This is a retrospective study of BMS patients over 5 years, when our outpatient clinic was established in November 2005 to October 2010. Fifty-nine BMS patients were listed by searching our outpatient database using the keywords tongue, mouth, glossodynia, glossitis, and BMS. Twenty patients were excluded: 12 patients who felt pain not on their tongues but on the oral mucous membrane or lips, 3 who felt numbness or an abnormal sensation on the tongue rather than pain, 1 who could not take the medication at all because his respiratory status grew worse due to lung cancer, 1 whose glossitis-like symptom was not a chief complaint but a secondary symptom and no longer was mentioned after the first consultation, and 3 who did not come back after the first consultation, including one who died a few days after the first consultation due to an accident. Consequently, 39 out of 59 BMS patients were diagnosed as suffering from primary glossodynia and appropriate for further investigation. All 39 patients had been diagnosed with primary glossodynia by a specialist such as a dentist and/or otorhinolaryngologist and, before consulting us, had already tried more than one of the conventional solutions such as NSAIDs; vitamin B complex; antidepressants including amoxapine, amitriptyline, paroxetine, and sertraline; anticonvulsants including gabapentin and clonazepam; antianxiety medicines; antipsychotics; pregabalin; and topical steroids, all of which had no effect or failed to have a sufficient effect. Most of the 39 patients stopped the conventional medications according to their own judgment before starting Kampo treatment, and, among the rest of the patients, those medications were not changed during the Kampo treatment. 

The 11-Point Numerical Rating Scale for Pain Intensity (NRSI) is a verbally administered scale that measures pain intensity (“how much pain do you feel right now?”) and is broadly used for the assessment of a wide variety of painful diseases. However, glossodynia patients often describe their pain mixed with unpleasantness, taste hypersensitivity, or other elusive sensations. Those symptoms, in addition, are prone to change into each other in a short while even during the treatment, which makes it difficult for patients to describe the improvement of symptoms merely in terms of comparing “pain” before and after the treatment. Therefore, all doctors defined the score 10 as corresponding to the severity of glossodynia before the start of the Kampo treatment and asked the patients to describe on a scale of 0–10 the severity of the symptoms remaining after the Kampo treatment, including unpleasantness, taste hypersensitivity, or other elusive sensations. In other words, a score of 10 meant that there was no change, a score of 5 meant that 50% of the severity of the symptoms due to glossodynia remained, and score of 0 meant that all symptoms caused by glossodynia were gone. 

Patients were regularly followed up on nearly every 4 weeks during the treatment. A Kampo prescription was regarded as effective when the patient's glossodynia remained improved for 2 or more consecutive consultations after each Kampo medication was started, and the score at the last consultation was utilized, whether a patient suddenly stopped attending the consultations by choice, changed his chief complaint and sought improvement for other symptoms rather than for glossodynia, or was still under treatment at the time.

Each Kampo medicine was prescribed as a decoction or as freeze-dried Kampo-extract granules according to each Kampo doctor's decision and/or each patient's preference. Decoction medicine was handmade at each patient's home following our clinic's protocol in which one day's herb mixture, dispensed at a pharmacy, was put in 600 mL of boiling water, was then boiled down to about 300 mL after 30~40 minutes, and this 300 mL was divided into 2 or 3 parts to be drunk 2 or 3 times throughout the day after the herbal residues were removed. The composition and herb doses for each decoction medicine are fixed by our school, whereas the extract granule medicines are manufactured, and the composition and herb doses of each extract granule medicine are fixed by pharmaceutical companies. The composition and herb doses of the 6 most effective Kampo prescriptions for glossodynia are described in Table 1. 

Data are expressed as *n* (%) or mean ± standard deviation (SD) in Tables 2 and 3. The following statistical tests were applied as appropriate: unpaired Student's *t*-test and chi-squared test with Fisher's exact test. A two-tailed *P* < 0.05 significance level was selected for all analyses. Microsoft Excel 2010 software was used for the computation of all statistical analyses.

This study received approval from the Human Research Ethics Committee of Chiba University Graduate School of Medicine (Identification no.: 1368).

## 3. Results

Reflecting that the use of complementary and alternative medicine (CAM) is most prevalent among women [6], 32 patients out of 39 (82.1%) were females, as most of our outpatients are, and there was no significant difference in age, average residual scores, or average onset of improvement between genders (see Table 2). The average duration of suffering from glossodynia was 107.2 ± 138.4 weeks, and the average duration of time spent on the conventional medication was 59.6 ± 88.1 weeks until the first consultation among all 39 patients (expressed as means ± standard deviation, resp.) (not shown in Tables). 

Twelve (30.8%) patients had no improvement (nonresponders), 27 (69.2%) patients had their severity of glossodynia lowered by 20% or more, and 23 (59.0%) patients showed a reduction of severity by half or more (high responders) out of a total of 39 patients. The average scores were decreased to 4.6 ± 4.2 from 10, and the average onset of improvement was 8.0 ± 7.7 weeks after starting Kampo treatment. The average latency to gain the max efficacy of Kampo treatment could not be calculated because 14 out of 39 patients were still in recovery as of March 1st, 2011. Those 14 patients' efficacy rates, gender difference, and average residual scores showed no significant change compared to the other patients (data not shown). 

Table 3 shows the comparison data between 12 nonresponders and 23 high responders whose scores decreased by 5 or more. There were no significant differences between the nonresponders and high responders in age, female/male ratio, and the rate of decoction. Not enough supplementary information was provided in clinical charts to compare nonresponders with high responders in mental aspects. The average drop-out time among the 12 nonresponders was 14.0 ± 15.9 weeks after starting Kampo treatment (data not shown), which is reasonable considering that the average onset of improvement was 8.0 ± 7.7 weeks after starting Kampo treatment (see Table 2). Five out of the 12 nonresponders, however, dropped out of Kampo treatment after their second consultation, which may have been too early to evaluate Kampo's effectiveness.

**Figure 1:**
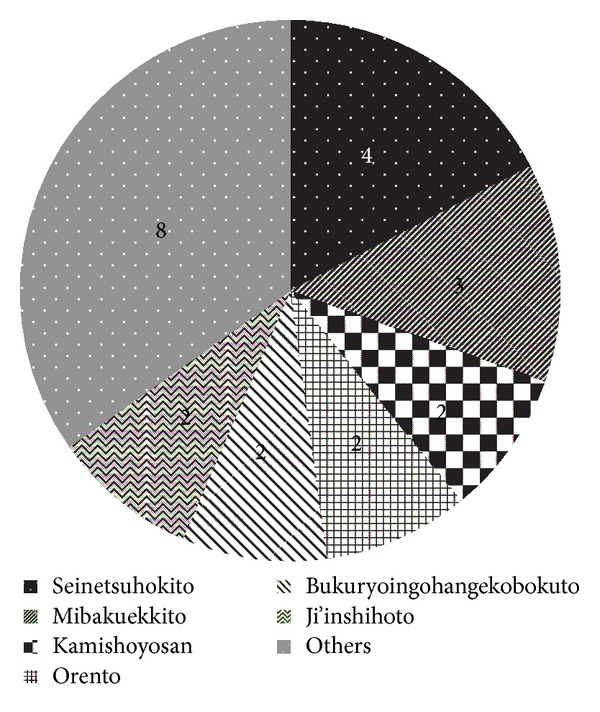
Effective Kampo prescriptions. The numbers show the patients per each Kampo prescription. Others: the combination of bakumondoto and daikenchuto, the combination of shosaikotokakikyosekko and tokakujokito, saikokeishikankyoto, bukuryoin, keihito, the combination of hachimigan and tokakujokito, kanzoshashinto, seishinrenshiin.

**Figure 2:**
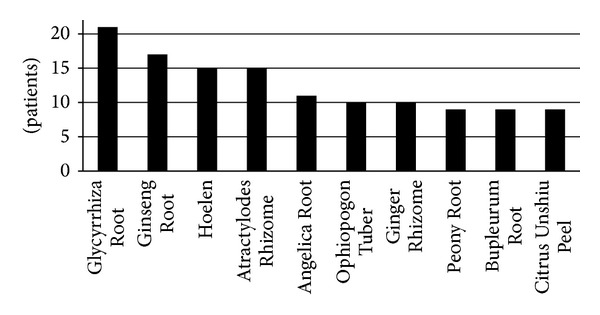
Ten herbs that overlapped most among effective Kampo prescriptions for glossodynia.

All Kampo prescriptions were chosen based on Kampo diagnoses made by Kampo experts.  Figure 1 shows the effective Kampo prescriptions in 23 high responders. Seinetsuhokito and mibakuekkito, both of which are decoction medicines used empirically for glossodynia in our school, were likely to be used more often than other medicines. However, all in all, a wide variety of Kampo prescriptions turned out to be effective for glossodynia, and there was no pattern among the effective Kampo prescriptions. Therefore, all herbs used in the 23 high responders were listed, and Figure 2 shows the 10 herbs that overlapped most among effective Kampo prescriptions. The most overlapped herb turned out to be Glycyrrhiza Root, also known as Licorice, which was used in 21 out of the 23 high responding patients. In addition, the top 4 most overlapped herbs, Glycyrrhiza Root, Ginseng Root (Panax Ginseng), Hoelen (Poria cocos Wolf), and Atractylodes (lancea) Rhizome, compose an essential Kampo prescription called “shikunshito”, a basic Kampo prescription from which many Kampo prescriptions derive. Shikunshito is prescribed to improve not only weakened gastrointestinal function but also hypofunction accompanied by decreased physical and mental strength such as in generalized fatigability [5]. 

## 4. Discussion

Conventional medical doctors introduce patients to Kampo experts when the Kampo medicines prescribed by them are not effective enough or when conventional medicine cannot improve patients' symptoms [7]. Quality control of the herbs used in Kampo has been established for both extract granules and decoctions, and the safety and reliability of Kampo have been well-established through the strict monitoring of side effects under the control of the Japanese Ministry of Health, Labor, and Welfare in the same way as conventional medicines are used [8]. In addition, the clinical efficacy of some Kampo medicines, especially daikenchuto and yokukansan, has been demonstrated in many clinical trials done by conventional medical doctors using the standard methods of conventional medicine [7, 9–11]. Our group has recently reported on other Kampo medicines which are remarkably effective in many cases refractory to conventional medicine [12–16]. The reliability and effectiveness of Kampo medicine thus promotes its integration with conventional medicine in Japan.

In our study, all patients had already tried one or more conventional treatments for glossodynia for a long time (59.6 ± 88.1 weeks on average, as described previously in Section 3) and showed no improvement, and they were consequently introduced to us or consulted us by themselves. After being treated with Kampo medicine, 69.2% of them exhibited a decrease in the severity of glossodynia of 20% or more, and 59.0% of them showed a reduction of severity by half or more, which demonstrates that Kampo medicine should be a valid alternative approach to refractory glossodynia. Compared to conventional treatments such as clonazepam [17], chlordiazepoxide [18], pregabalin [19], and SSRIs [20, 21], the effective rate of Kampo medicine for glossodynia could be superior, because we treated only refractory glossodynia cases that had already tried conventional medicine. However, it might be meaningless to compare the efficacy rate between conventional treatment and Kampo medicine because Kampo medicine can still function as a valid alternative approach for patients whose glossodynia remains incurable even after all conventional treatments have been tried. 

Glossodynia has female predominance (female-to-male ratio of 3 : 1 to 7 : 1) [22], and we found that female glossodynia patients may need more time until the onset of improvement than male patients (see Table 2). Although this result must be confirmed in a larger population, this tendency may be because glossodynia is often caused by a long history over which vital energy deficiency and blood deficiency are developed, and women tend to have a more serious and longer history of such deficiencies after menopause than men. 

There was no significant difference in the rate of decoction between nonresponders and high responders, although Kampo decoction medicines are often expected to work with more effectiveness than extract granule medicines (see Table 3). In other words, the effectiveness of extract granule prescriptions is equal to that of decoction medicines as long as the choice of prescription is appropriate to each case based on Kampo diagnosis. More than 60% of high responders, in fact, were successfully treated with extract granule prescriptions (see Table 3). Whether in the form of extract granules or decoction, Kampo medicine employs a much smaller amount (1/2~1/10) of herbs than other Asian traditional herbal medicines while realizing the high quality and efficacy of the treatment (see Table 1). This characteristic of Kampo medicine contributes greatly to the protection of rare plants. 

Seinetsuhokito was used most frequently among effective prescriptions (see Figure 1), which was consistent with the previous report written in Japanese that the first choice for glossodynia is seinetsuhokito in Kampo medicine [23]. Kamishoyosan, one of the third most frequently used prescriptions in our current study, is used most frequently for glossodynia in another report written in Japanese [24]. All in all, however, there was almost no pattern among effective Kampo prescriptions, which implies that it may be difficult for non-Kampo experts to choose an appropriate Kampo medicine for each glossodynia patient. This is not surprising because Kampo prescriptions by nature are chosen based on Kampo-specific diagnosis regardless of conventional diagnosis, which means that different Kampo prescriptions are effective as long as each patient has a tailored Kampo diagnosis even if patients have the same conventional diagnosis. However, we tried to find any common tendency in Kampo philosophy among glossodynia patients and found that 10 herbs, which overlapped most among effective prescriptions for glossodynia in our current study, indicate a certain solution for glossodynia (see Figure 2). 

Glycyrrhiza Root, the herb that overlapped most among the effective treatments, is thought to harmonize the effects of the other constituent herbs without diminishing their characteristics, and it is contained by more than 70% of Kampo prescriptions [5]. The frequency of its use in our study (21 out of 23 patients) was clearly higher than 70%, suggesting that Glycyrrhiza Root alone has some ameliorating efficacy in glossodynia. Accumulating studies have revealed that Glycyrrhiza has an anti-inflammatory effect [25] and, interestingly, a randomized, double-blind, placebo-controlled trial shows that a patch containing extract of Glycyrrhiza Root is significantly effective in treating recurrent aphthous ulcers [26], which also strongly indicates that Glycyrrhiza Root alone can be effective for an inflammatory symptom appearing inside the mouth. 

Ginseng Root was the second most overlapped herb among effective prescriptions for glossodynia in our current study (see Figure 2). Ginseng Root is one of the most well-known herbs in the world and ranks as a frequently used herbal remedy in Europe [27]. The therapeutic efficacy of Ginseng Root in painful diseases such as fibromyalgia [28] is still controversial, although Ginseng Root was lately revealed to moderate the immune response in a systematic review [29] and to potentially have anti-inflammatory and analgesic effects in several animal studies [30, 31]. Both Hoelen and Atractylodes Rhizome were the third most overlapped herb among effective prescriptions for glossodynia (see Figure 2). Hoelen has been traditionally used for promoting urination and reducing edema in clinical practice, and actually recent researches show that Hoelen has renoprotective effects by modulating water balance [32, 33]. Atractylodes Rhizome not only has a diuretic effect similar to Hoelen by suppressing water reabsorption in the kidney [34] but also a randomized pilot study showed that Atractylenolide I, extracted from Atractylodes Rhizome, improves appetite and performance status in gastric cancer cachexia patients [35]. 

Generally, it is regarded as meaningless to list the overlapping herbs used by high responders and to assert an individual herb's function, because Kampo prescriptions are thought to exert their effectiveness as a function of the herb combination. However, our most important finding in our current study is that the top 4 overlapping herbs form a specific Kampo prescription, called “shikunshito”. Shikunshito is one of the most well-known and basic Kampo prescriptions and is used to improve deficiencies of vital energy (“Ki” in Kampo medicine and “Qi” in Chinese) and deficiencies of digestive function in Kampo medicine [5, 36]. In fact, an old Japanese medical book “Kohouyakugi” written in 1894 by Sohaku Asada, a famous Kampo doctor, reported that Ginseng Root, Hoelen, and Atractylodes Rhizome exert a common efficacy in recovering the gastrointestinal function, although each herb also has many other differing therapeutic effects. This common ameliorating effect of Ginseng Root, Hoelen, and Atractylodes Rhizome on the gastrointestinal function, when taken together with other differing therapeutic efficacies of Glycyrrhiza Root, Ginseng Root, Hoelen, and Atractylodes Rhizome, reenergizes patients with a declined physical and mental status, which is the core therapeutic efficacy of shikunshito. A different group reported 5 cases that were successfully treated with Kampo medicine, and shikunshito-containing prescriptions were used in 3 out of the 5 cases [37], and, in our current study, in fact, 17 out of 23 high responders took prescriptions including 3 or more herbs which compose shikunshito. 

Although our current study suggests that shikunshito has a key role, a single shikunshito prescription may not improve glossodynia on its own. The other overlapping herbs should be concomitantly effective for glossodynia because they also play important roles, according to Kampo philosophy. Ginger Rhizome is usually decocted with shikunshito and improves the digestive system; a finding that is supported by accumulating reports showing its effectiveness for chemotherapy-induced, pregnancy-induced, and postoperative nausea and vomiting [38, 39]. Angelica Root has several subspecies, and one of them, Angelica sinensis (dong quai in Chinese), which is the most well-known Angelica used also in other CAMs, is thought to treat female menstrual ailments, nourish blood, and invigorate vital energy in Chinese medicine [40]. Angelica sinensis, as a matter of fact, is proven to increase sexual activity and influence fertility in animal models, although human studies could not support this finding [41]. Japanese Angelica, which is used in Kampo medicine, has a reputation for its safety and quality control [42] and is thought to have a similar function to Angelica sinensis, although its pharmacological mechanism is not scientifically elucidated in detail yet. Ophiopogon Tuber is thought to have an anti-inflammatory effect and to treat dry cough and dry mouth, tongue, and throat by nourishing and moistening the lungs and the bowels [40]; a finding that is supported by the reports that Ophiopogon has anti-inflammation activity [43] and has a beneficial effect on a mouse model for Sjogren's syndrome [44] and on an epithelial injury model for studies of mucociliary transport [45]. Peony Root is often concurrently used with Angelica Root and improves blood deficiency, dissipates blood stagnation, and relieves pain [5, 36, 40]. Shakuyakukanzoto, a Kampo prescription consisting of Glycyrrhiza Root and Peony Root, has been well-known among primary care doctors in Japan for having an immediate relieving effect on muscle cramps [5, 36], which has been proven by several open-labeled trials in hemodialysis patients [46, 47]. Shakuyakukanzoto also has a significant suppressive effect on duodenal spasms during endoscopic retrograde cholangiopancreatography [48]. Taken together, the combination of Glycyrrhiza Root and Peony Root seems to have an antispasmodic pain-relieving effect on both skeletal and smooth muscle. In addition, Paeoniflorin, one of the main ingredients in Peony extract, has a protective effect on its own on gastric mucosal injury [49]. Bupleurum Root is an important herb in Kampo medicine, and it forms a “Bupleurum” prescription category that is used for specific pathological conditions such as alternating chills and fever, fullness and a choking feeling in the chest and hypochondriac region, and stress-induced imbalance [5, 36]. Accumulating reports have revealed that Bupleurum has immunomodulatory, antiinflammatory, antiviral, and anti-ulcer activities [50] as well as an antidepressant-like effect in animal depression models [51, 52]. Citrus Unshiu Peel has a releasing action for Ki regurgitation, whereas Bupleurum Root has an obstruction-removing action for Ki when there is obstruction [5], which means that both have ameliorating effects on mental imbalance. In summary, glossodynia is a complicated pathological state caused not only by sustained vital energy deficiency and/or blood deficiency but also by mental imbalance from the viewpoint of Kampo philosophy. Kampo medicine for glossodynia, therefore, should be carefully chosen among prescriptions including shikunshito by considering which deficiency or imbalance is most necessary to improve depending on the case. In addition, it would be intriguing to make a new antiglossodynia Kampo prescription consisting of these top 10 overlapping herbs.

## 5. Conclusions

We have herein reported our single-institution 5-year experience treating refractory glossodynia with Kampo medicine. Kampo treatment had beneficial effects on 69.2% of refractory glossodynia patients, and the top 4 overlapping herbs used to treat the responders are the components of a well-known Kampo prescription, shikunshito. Thus, Kampo medicine showed valid efficacy, even in cases in which glossodynia remained incurable after conventional treatments were tried. Although more research is required to further clarify the effectiveness of Kampo medicine, these findings confirm that Kampo medicine is a promising alternative for treating refractory glossodynia.

## Figures and Tables

**Table 1:** Composition and herb doses of the 6 most effective Kampo prescriptions for glossodynia.

Seinetsuhokito	
Ginseng Root (3), Angelica Root (3), Peony Root (3), Ophiopogon Tuber (3),	
Atractylodes Rhizome (3.5), Hoelen (3.5), Cimicifuga Rhizome (1),	
Schisandra Fruit (1), Scrophularia Buergeriana Root (1), and Glycyrrhiza Root (1)	
Mibakuekkito	
Astragalus Root (4), Glycyrrhiza Root (1.5), Jujube Fruit (2), Ginseng Root (4),	
Atractylodes Rhizome (4), Ginger Rhizome (1), Angelica Root (3),	
Citrus Unshiu Peel (2), Cimicifuga Rhizome (0.5), Bupleurum Root (2),	
Schisandra Fruit (2), and Ophiopogon Tuber (5)	
Kamishoyosan	
Bupleurum Root (3), Peony Root (3), Atractylodes Lancea Rhizome (3),	
Angelica Root (3), Hoelen (3), Gardenia Fruit (2), Moutan Bark (2),	
Glycyrrhiza Root (1.5), Ginger Rhizome (1), and Mentha Herb (1)	
Orento	
Pinellia Tuber (6), Coptis Rhizome (3), Glycyrrhiza Root (3), Cinnamon Bark (3),	
Jujube Fruit (3), Ginseng Root (3), and Ginger Rhizome (3)	
Bukuryoingohangekobokuto	
Pinellia Tuber (6), Hoelen (5), Atractylodes Lancea Rhizome (4), Magnolia Bark (3),	
Citrus Unshiu Peel (3), Ginseng Root (3), Perilla Herb (2), Immature Orange (1.5),	
and Ginger Rhizome (1)	
Ji'inshihoto	
Cyprus Rhizome (3), Bupleurum Root (3), Peony Root (3), Anemarrhena Rhizome (3),	
Citrus Unshiu Peel (3), Angelica Root (3), Ophiopogon Tuber (3),	
Atractylodes Rhizome (3), Hoelen (3), Glycyrrhiza Root (1), Mentha Herb (1),	
Lycium Bark (3), and Fritillary Bulb (2)	

Seinetsuhokito and mibakuekkito are decoction medicines, and kamishoyosan, orento, bukuryoingohangekobokuto, and ji'inshihoto are extract-granule medicines. The numbers in the round brackets show the one-day dose of each herb (grams).

**Table 2:** Patient demographic characteristics.

	Total (*n* = 39)	Female (*n* = 32)	Male (*n* = 7)	*P* value (Female versus male)
Age (years)	65 ± 9	64.3 ± 8.8	70 ± 6.7	NS^#^
No improvement (nonresponders)	12 (30.8%)	9 (28.1%)	3 (42.9%)	NS*
Decreased scores ≦ 2	27 (69.2%)	23 (71.9%)	4 (57.1%)	NS*
Average residual scores	4.6 ± 4.2	4.6 ± 4.1	4.8 ± 4.9	NS^#^
Average onset of improvement (weeks)	8.0 ± 7.7	8.4 ± 8.3	5.8 ± 3.5	NS^#^
Decreased scores ≦ 5 (high responders)	23 (59.0%)	19 (59.4%)	4 (57.1%)	NS*

Data are presented as *n* (%) or mean ± SD.

^#^ *t*-test, *chi-squared test.

NS: no significance.

**Table 3:** Comparison between nonresponders and high responders.

	Nonresponders (*n* = 12)	High responders (*n* = 23)	*P* value
Age (years)	65.8 ± 7.6	64.8 ± 9.7	NS^#^
Female/male	9 (75%)/3 (25%)	19 (82.6%)/4 (17.4%)	NS*
Rate of decoction	5 (41.7%)	9 (39.1%)	NS*

Data are presented as *n* (%) or mean ± SD.

^#^ *t*-test, *chi-squared test.

NS: no significance.
